# CRISPR/Cas9-Mediated Genome Editing in Soybean Hairy Roots

**DOI:** 10.1371/journal.pone.0136064

**Published:** 2015-08-18

**Authors:** Yupeng Cai, Li Chen, Xiujie Liu, Shi Sun, Cunxiang Wu, Bingjun Jiang, Tianfu Han, Wensheng Hou

**Affiliations:** 1 Ministry of Agriculture Key Laboratory of Soybean Biology (Beijing), Institute of Crop Sciences, Chinese Academy of Agricultural Sciences, Beijing, 100081, China; 2 National Center for Transgenic Research in Plants, Institute of Crop Sciences, Chinese Academy of Agricultural Sciences, Beijing, 100081, China; Mayo Clinic, UNITED STATES

## Abstract

As a new technology for gene editing, the CRISPR (clustered regularly interspaced short palindromic repeat)/Cas (CRISPR-associated) system has been rapidly and widely used for genome engineering in various organisms. In the present study, we successfully applied type II CRISPR/Cas9 system to generate and estimate genome editing in the desired target genes in soybean (*Glycine max* (L.) Merrill.). The single-guide RNA (sgRNA) and Cas9 cassettes were assembled on one vector to improve transformation efficiency, and we designed a sgRNA that targeted a transgene (*bar*) and six sgRNAs that targeted different sites of two endogenous soybean genes (*GmFEI2* and *GmSHR*). The targeted DNA mutations were detected in soybean hairy roots. The results demonstrated that this customized CRISPR/Cas9 system shared the same efficiency for both endogenous and exogenous genes in soybean hairy roots. We also performed experiments to detect the potential of CRISPR/Cas9 system to simultaneously edit two endogenous soybean genes using only one customized sgRNA. Overall, generating and detecting the CRISPR/Cas9-mediated genome modifications in target genes of soybean hairy roots could rapidly assess the efficiency of each target loci. The target sites with higher efficiencies can be used for regular soybean transformation. Furthermore, this method provides a powerful tool for root-specific functional genomics studies in soybean.

## Introduction

Soybean (*Glycine max* (L.) Merrill.) is an ancient polyploid and important legume crop with great economic value. Soybean provides abundant protein and oil for food production and animal forage. In recent years, a variety of genetic approaches have been used to improve the agricultural traits of soybean. However, several bottlenecks hinder this progress. Current efforts for soybean gene function research include overexpression and RNAi (RNA interference)-based approaches. *A*. *tumefaciens*-mediated transformation has been widely used to generate transgenic soybean plants [1, 2]. However, there are some disadvantages to this traditional method. The foreign genes (DNA fragments) are typically randomly integrated into the plant genome after entering the nucleus, which may generate negative results, such as the disruption of plant endogenous genes or exogenous gene silencing [3]. RNAi might silence entire gene families, although the silencing of an individual target gene is desired [4].

The genome of soybean is complex because these genes are highly duplicated [5, 6]. Thus, precise and straightforward methods for researching gene functions and genome engineering are required. Recently, CRISPR/Cas9 has emerged as a robust and effective technology for editing each member of a gene family without influencing other genes or simultaneously editing multiple genes of interest, thereby overcoming the shortcomings of the traditional methods described above. Targeted genome modifications induced using this new system have been successfully introduced into many plant species, including many major crops, such as rice [7–10], wheat [11], sorghum [9] and maize [12]. Thus, this technology might be used to examine soybean gene functions.

The main characteristic of CRISPR/Cas9 system is the Cas9 protein, which comprises two functional domains: the RuvC-like domain and the HNH nuclease domain [13]. The endonuclease Cas9 can be guided by a synthetic single-guide RNA to recognize target sequences and produce double strand breaks (DSBs) at desired target sites [13, 14]. DSBs subsequently cause a series of complex DNA self-repair mechanisms in the cell and generate various site-specific genetic alterations through non-homologous end joining (NHEJ) or homology-directed repair (HDR). The NHEJ pathway is error-prone and typically generates insertions or deletions (indels) within the target sequence. When these indels introduce a frameshift mutation or disrupt important functional domains, the functions of the target genes will be damaged [7, 15, 16]. The possibility of homologous recombination will significantly increases in the present of homologous DNA fragments during the repair process. In addition, the recombination efficiency caused by double strand breaks can be improved by one thousand-fold [17]. According to this principle, the HDR pathway can be used to induce precise gene targeting using a homologous donor DNA as a template, such as the introduction of specific point mutations or the insertion of desired sequences in the target locus [7, 18]. The sequence of a fragment could also be replaced using an exogenous donor template [19].

It is simple to engineer the CRISPR/Cas9 vector because only sgRNA need to be customized for different genomic sites and because the Cas9 protein is codon-optimized, with no need for reconstructing every genome modification [20]. Therefore, the CRISPR/Cas9 system is a straightforward, economical and efficient genome editing technology. However, to date, this technology has not been widely used in soybean.

There are many disadvantages to the *A*. *tumefaciens*-mediated transformation of soybean [21], such as its low transformation frequency and long procedure. Additionally, this method is labor-intensive and requires proficient skills. Thus, *Agrobacterium rhizogenes*-mediated transformation has provided an alternative method for gene function research in soybean. This system transfers T-DNA from both the Ri plasmid of *Agrobacterium rhizogenes* and the binary vector designed to the genome to generate hairy roots upon wounding [22]. *Agrobacterium rhizogenes*-mediated transformation has also been employed to detect targeted mutations induced through zinc-finger nucleases in soybean hairy roots [23]. Therefore, we considered that hairy root transformation using *Agrobacterium rhizogenes* combined with the CRISPR/Cas9 system will provide a rapid and efficient method for gene function investigation in soybean. In the present study, we examined whether the CRISPR/Cas9 system could be used to edit endogenous or exogenous genes in soybean hairy roots. There has been previous similar study [24]. Assessing the effectiveness of target sites in hairy roots prior to whole-plant transformation could resolve the problem of the traditional transformation techniques being too labor-intensive and inefficient to be useful on a large scale. We also examined the capacity of CRISPR/Cas9 for simultaneous knockout of multiple genes using one sgRNA to develop a technique for testing the functions of gene families.

## Materials and Methods

### Plant Materials

The T6 generation transgenic soybean line (genotype, *Zigongdongdou*) harboring a homozygous *bar* transgene and a wild type soybean line (genotype, *Williams 82*) were used.

### SgRNA Design and Construction of SgRNA: Cas9 Expression Vector

To construct a plasmid vector expressing Cas9 and sgRNA simultaneously, the Cas9 gene sequence was codon-optimized for dicotyledons and placed downstream of the maize ubiquitin promoter together with customized sgRNA driven by the *Arabidopsis* U6 promoter. GFP driven by the CaMV 35S promoter was used for the rapid visual screening of transgenic hairy roots. These services were ordered from ViewSolid Biotech (Beijing). We designed these sgRNAs using the web-based tool CRISPR-P (http://cbi.hzau.edu.cn/crispr/) [25]. The sequences and other data of the analyzed soybean endogenous genes were downloaded from phytozome v9.1 (www.phytozome.net/). These sequences were subjected to analysis using the CRISPR-P web tool, which highlighted all potential CRISPR sgRNA sequences (20 bp) immediately followed by 5’-NGG (PAM) in the forward and reverse strands. We selected sequences in which the first base was a guanine (G) nucleotide. When the first base of the sgRNA sequence is not G, an extra G can alternatively be appended before the 5’ end of the sequence of sgRNA [26]. DNA sequences encoding sgRNA designed to target seven specific sites were used in the present study. For each target loci, a pair of DNA oligos were synthesized from BGI (Beijing) and annealed to generate dimers. These dimers were subsequently ligated upstream of the sgRNA scaffolds in the plasmid vector simultaneously expressing Cas9 and sgRNA. After transformation into *E*. *coli* DH5α, the consequent constructs were purified using the TIANprep Rapid Mini Plasmid Kit (TIANGEN) for subsequent use in soybean hairy root transformation.

### Hairy Root Transformation Using *A*. *rhizogenes* K599

The plasmid vector used for expressing Cas9 and single-guide RNA was mobilized into *A*. *rhizogenes* K599 via electroporation. The hypocotyl and cotyledonary node of the T6 generation transgenic soybean line (genotype, *Zigongdongdou*) harboring a homozygous *bar* transgene and wild type soybean line (genotype, *Williams 82*) were used for tissue culture and transformation with slight modifications from the protocol previously described [27]. Briefly, smooth and plump soybean seeds were selected and surface sterilized using chlorine for 16~20 h. Subsequently, the seeds were cultivated on pre-prepared solid mediums containing 3.10 g/L basal salt mixture (PhytoTechnology Laboratories), 20 g/L sucrose (Sigma), pH 5.8, and 7 g/L agar (Sigma) at 25°C (16 h of light/8 h of darkness) for 5 days. *A*. *rhizogenes* K599 containing plasmid vectors were activated twice. Initially, some single bacterial colonies were inoculated in fresh liquid YEP medium (20 ml) containing 50 mg/L kanamycin, shaken (200 rpm) at 28°C overnight and subsequently diluted with 1:1000 fresh liquid YEP medium, followed by rocking at 28°C until the OD_600_ was 0.6~0.8 [28]. Cotyledons with approximately 5 mm hypocotyl of the 5-day pre-cultured soybean seeds were used as explants. The hypocotyl and cotyledonary nodes of these explants were softly wounded with a scalpel and immersed into activated *A*. *rhizogenes* K599 solution for 30 min. After infection, the explants were placed onto filter paper above co-cultivation medium (1/10 MS, 30 g/L sucrose, 3.9 g/L MES, PH 5.4, 7 g/L agar, 154 mg/L dithiothreitol, 100 μM/L acetosyringone) and grown in darkness at 22°C for 5 days to facilitate T-DNA transfer from the plasmid into the cells. The explants were subsequently rinsed approximately four times with liquid medium containing 1/2 MS, 30 g/L sucrose, 0.6 g/L MES, PH 5.8, 250 mg/L cefotaxime and 250 mg/L carbenicillin and transferred onto medium containing 1/2 MS, 30 g/L sucrose, 0.6 g/L MES, PH 5.8, 7 g/L agar, 250 mg/L cefotaxime and 250 mg/L carbenicillin at 28°C. After cultivation for two weeks, hairy roots harboring the desired binary vector were identified for further analysis.

### Characterization of Transgenic Hairy Roots Harboring SgRNA: Cas9

Hairy roots grown to a length of 5 to 6 cm were marked with numbers and screened using a dissecting fluorescence microscope (Nikon SMZ1500), and the transgenic roots showed GFP fluorescence labeling (S6 Fig).

### Screening for Mutations Induced through the CRISPR/Cas9 System in Target Genes

The genomic DNA of transgenic roots was extracted using the CTAB method. PCR primers were designed to amplify a 400~800 bp amplicon containing the target sequence. CRISPR/Cas9-induced mutagenesis involves the introduction of small insertions or deletions (indels) into target sequences. We adopted two strategies for detecting CRISPR/Cas9-induced mutations: PCR/RE and T7EI assays. PCR/RE assays were used for *bar*-SP1, *GmFEI2*-SP1, *GmFEI2*-SP2, *GmFEI2*-SP3 and *GmSHR*-SP3 which have an adaptive restriction enzyme site at the Cas9 cleave site. The mutational PCR products cannot be digested with restriction enzymes that recognize wild-type sequences, and produced undigested bands. These undigested bands were purified using the TIANgel Midi Purification Kit (TIANGEN) according to the manufacturer’s instructions and further characterized through subcloning and sequencing. Indel frequencies were calculated by measuring the band intensities using the ImageJ gel quantification software. Each lane was independent, and each band was measured according to the intensity after subtracting the background. The indel frequencies were calculated using the formula: indel (%) = 100 × a / (a + b + c), where a is the intensity of the undigested band and b and c are the intensities of the two digested bands [20]. T7EI assays were used for *GmSHR*-SP1 and *GmSHR*-SP2 which has no appropriate restriction enzyme site in the target sequences. PCR primers were designed to amplify 400~800 bp fragments spanning the genomic target site. The PCR products were denatured and renatured using PCR to generate heteroduplexes. The reaction products were subsequently digested with T7EI nuclease and subsequently analyzed using 2.0% (wt/vol) agarose gel electrophoresis. The annealed heteroduplexes were cleaved with T7EI, whereas the wild type and mutant homoduplexes remained intact. The mutant amplicons were subcloned and sequenced to identify the mutations at target sites. The mutation frequencies were estimated using a previously described formula [20, 26]. All specific primers used in the present study are shown in S1 Table.

## Results

Target Site Selection and Construction of the SgRNA: Cas9 Expression Vector

**Fig 1:**
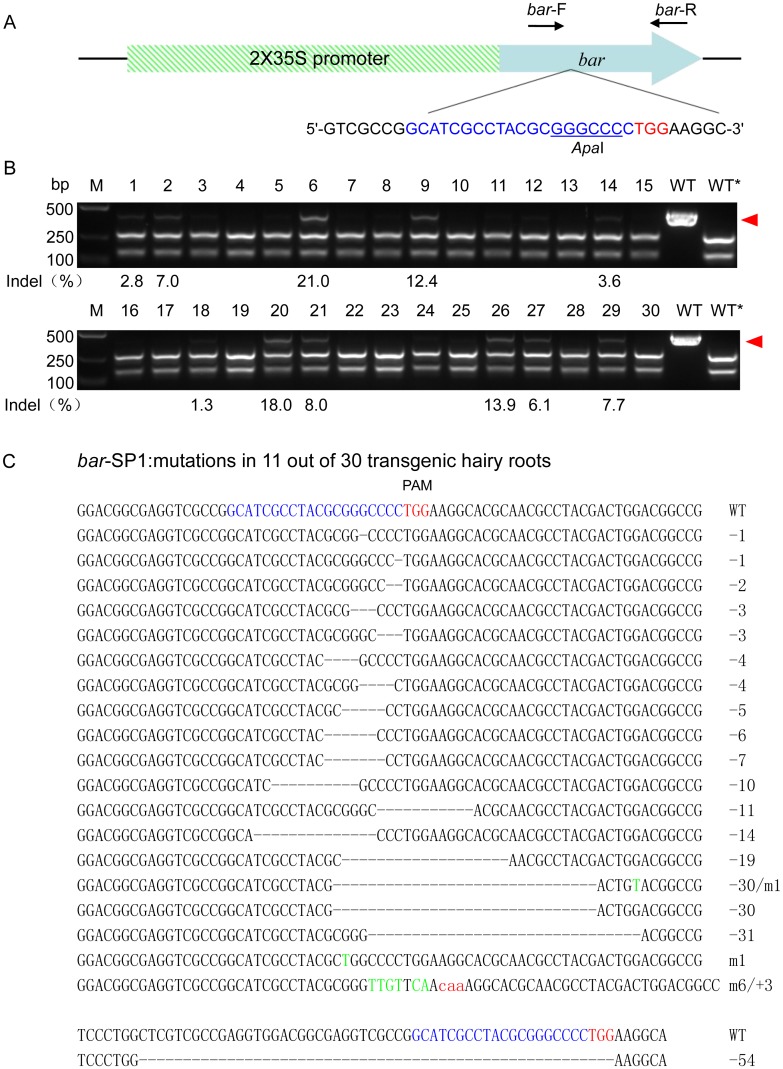
CRISPR/Cas9-induced mutations at a transgene *bar* in soybean hairy roots. (A) Schematic illustrating the targeted *bar* sequence (blue) and corresponding PAM (red). PCR amplification spanning the target loci was conducted using the primers *bar*-F and *bar*-R. Restriction enzyme *Apa*I at the Cas9 cleavage site is underlined. (B) PCR/RE assay to detect CRISPR/Cas9-induced mutations in target loci. Lanes 1–30, PCR products of samples treated with sgRNA: Cas9 were digested with *Apa*I. Lanes WT and WT*, undigested and digested wild-type controls, respectively. The red arrowhead indicates the undigested bands. The numbers at the bottom of the gels indicate mutation frequencies measured according to band intensities. M, DL2000 ladder DNA marker. (C) Cloning and sequencing of the undigested bands. Deletions and insertions are indicated as dashes and red lowercase letters, respectively. Base substitutions are indicated with green capital letters. The types of indels are indicated in the right column.

**Fig 2:**
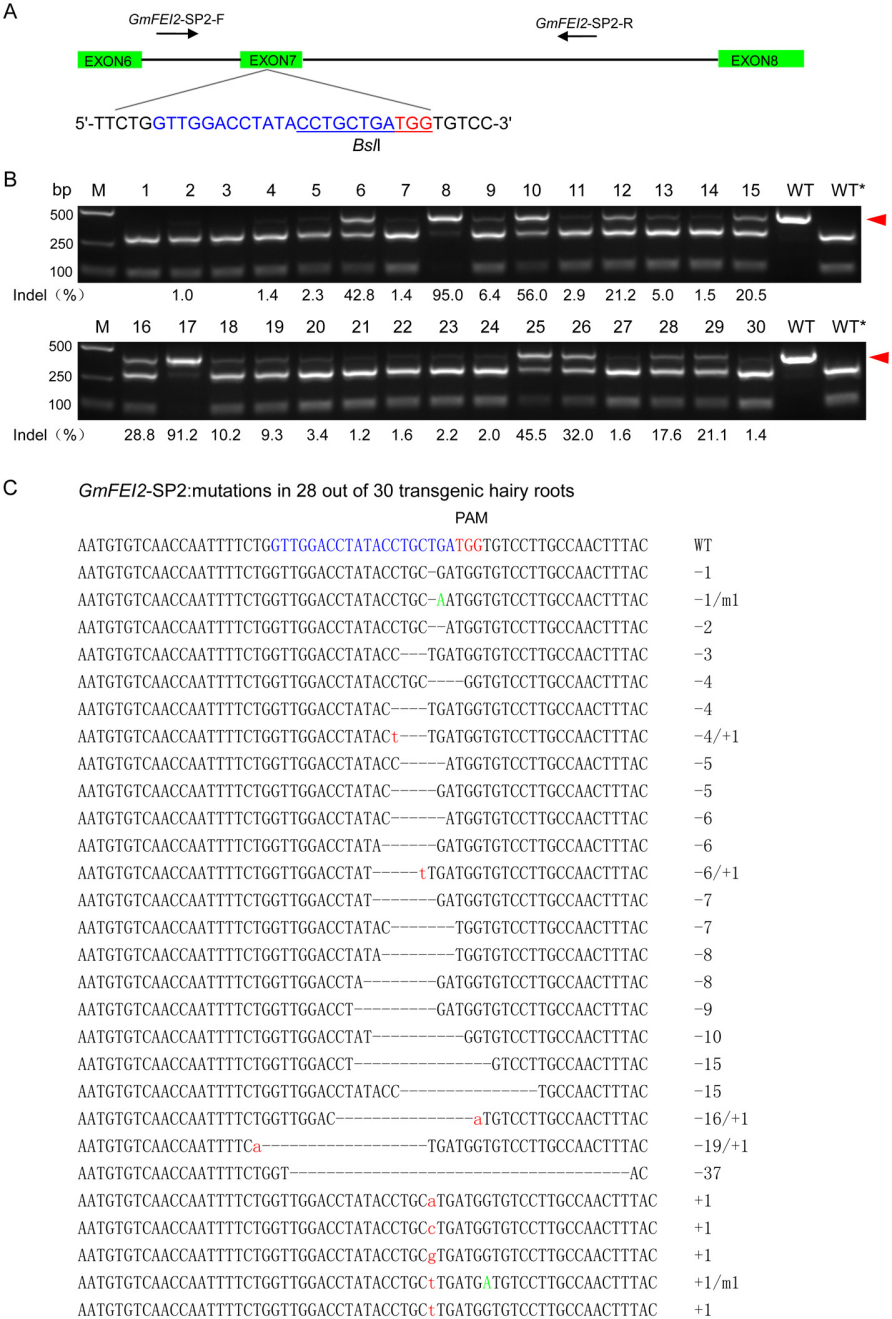
CRISPR/Cas9-induced mutations in the *GmFEI2*-SP2 target site. (A) Schematic illustrating the *GmFEI2*-SP2 target sequence (blue) and corresponding PAM (red). PCR amplification spanning the target loci was conducted using the primers *GmFEI2*-SP2-F and *GmFEI2*-SP2-R. The restriction enzyme *Bsl*I at the Cas9 cleave site is underlined. (B) PCR/RE assay to detect CRISPR/Cas9-induced mutations in target loci. Lanes 1–30, PCR products of samples treated with sgRNA: Cas9 were digested with *Bsl*I. Lanes WT and WT*, undigested and digested wild-type controls, respectively. The red arrowhead indicates the undigested bands. The numbers at the bottom of the gels indicate mutation frequencies measured according to band intensities. M, DL2000 ladder DNA marker. (C) Cloning and sequencing of the undigested bands. Deletions and insertions are indicated as dashes and red lowercase letters, respectively. Base substitutions are indicated with green capital letters. The types of indels are indicated in the right column.

**Fig 3:**
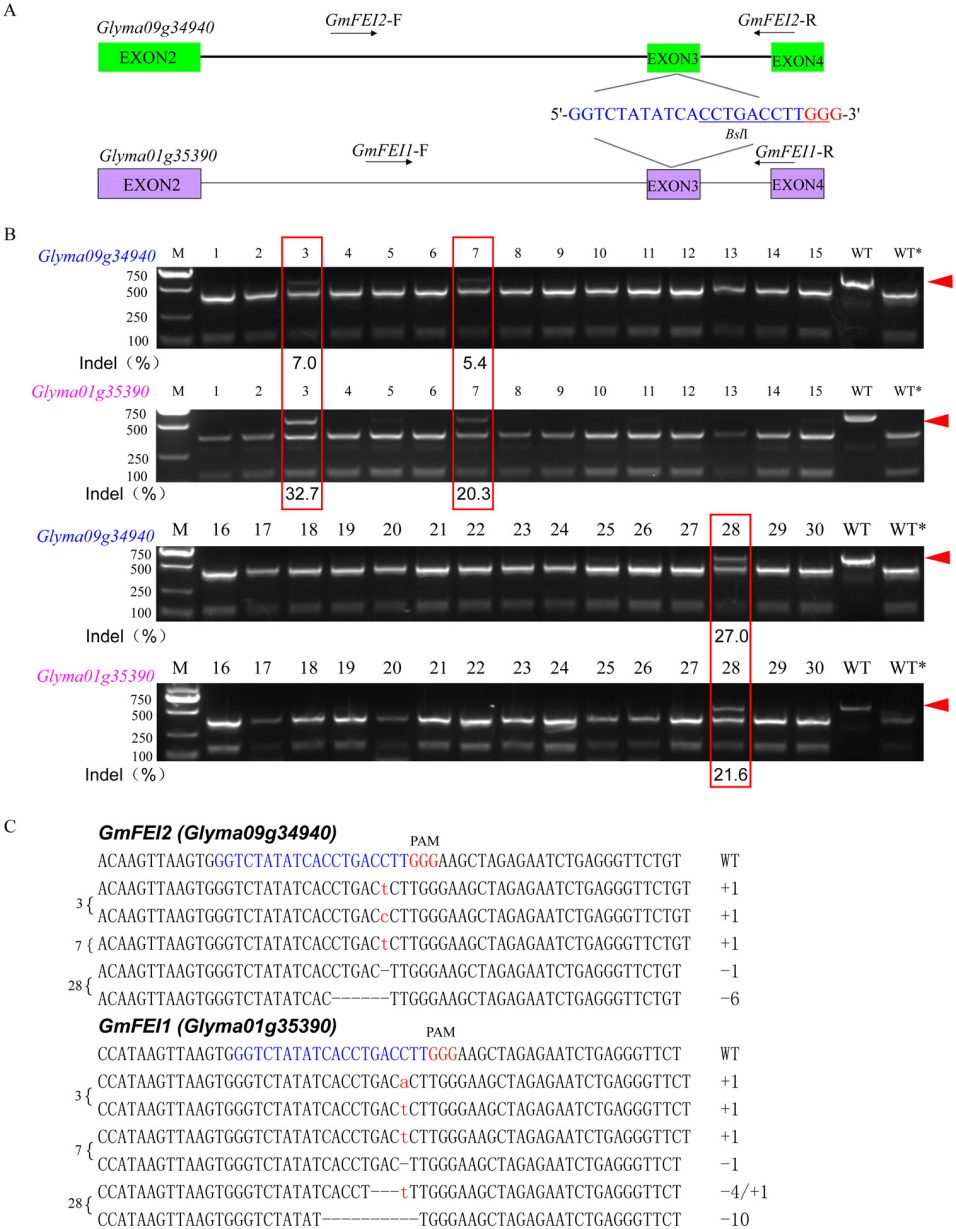
Simultaneous edition of two target sites with only one customized sgRNA. (A) Schematic illustrating the target sequence (blue) and corresponding PAM (red). PCR amplifications spanning the target loci were conducted from an identical DNA template using the specific pair primers *GmFEI2*-F/*GmFEI2*-R and *GmFEI1*-F/*GmFEI1*-R, respectively. Restriction enzyme *Bsl*I at the Cas9 cleavage site is underlined. (B) PCR/RE assay to detect CRISPR/Cas9-induced mutations in target sites of the two genes. Lanes 1–30, PCR products from the samples treated with sgRNA: Cas9 were digested with *Bsl*I. Lanes WT and WT*, undigested and digested wild-type controls, respectively. The red arrowhead indicates the undigested bands. The numbers at the bottom of the gels indicate mutation frequencies measured according to band intensities. M, DL2000 ladder DNA marker. The red frame indicates two simultaneous gene-editing events occurring in the same hairy root. (C) Cloning and sequencing of the undigested bands. Deletions and insertions are indicated as dashes and red lowercase letters, respectively. The types of indels are indicated in the right column. The numbers before brackets correspond to the numbers of the lanes.

To extensively explore whether the CRISPR/Cas9 system can generate double strand breaks (DSBs), which can lead to genome modifications at desired target sites of soybean exogenous genes and/or endogenous genes, a list of genes were tested in soybean hairy roots, such as the homozygous transgene (*bar*) and three soybean endogenous genes [*GmFEI1*(*Glyma01g35390*), *GmFEI2*(*Glyma09g34940*), and *GmSHR*]. We designed a single sgRNA, namely *bar*-SP1, to target the homozygous *bar* transgene (Fig 1A) and three sgRNAs, *GmFEI2*-SP1, *GmFEI2*-SP2 and *GmFEI2*-SP3, which target different sites of the soybean endogenous gene *GmFEI2* (S1A Fig, Figs 2A and 3A). We also designed three sgRNAs, *GmSHR*-SP1, *GmSHR*-SP2 and *GmSHR*-SP3, which target different sites of the soybean endogenous gene *GmSHR* (S2A, S3A and S4A Figs). To determine whether this technology could simultaneously disrupt two endogenous genes with only one customized sgRNA, we used the sgRNA *GmFEI2*-SP3 to simultaneously target the third exon of both *GmFEI1* and *GmFEI2* (Fig 3A).

A vector expressing both sgRNA and Cas9 was constructed (S5 Fig). Cas9 was codon-optimized for dicotyledons and induced through the maize ubiquitin promoter. The sgRNA cassette was induced through the *Arabidopsis* U6 promoter. Green fluorescent protein (GFP) induced through a cauliflower mosaic virus (CaMV) 35S promoter was used as a visible marker to rapidly and easily screen targeted events.

### CRISPR/Cas9-induced Mutations in the *bar* Transgene

A summary of the CRISPR/Cas9 genome editing assays used in the present study is shown in Table 1. To determine whether the CRISPR/Cas9 system induces mutations at the target sites of exogenous genes, we customized a sgRNA targeting the exogenous gene *bar* in soybean hairy roots. The T6 transgenic soybean line (genotype, *Zigongdongdou*) harboring a homozygous *bar* transgene with a *Apa*I recognition site within the CRISPR/Cas9 target sequence was generated. PCR/restriction enzyme (PCR/RE) assays were utilized to detect mutations in the target locus. Undigested bands were detected in 11 of the 30 transgenic hairy roots. The results showed that the mutation frequencies ranged from 1.3% to 21.0%, based on the band intensities (Fig 1B). Undigested products were subsequently cloned and sequenced to detect the type of indels in the targeted *bar* gene. Small insertions and deletions were detected in the target site (Fig 1C).

**Table 1:** Summary of CRISPR/Cas9 genome editing assays in the present study.

Target gene	Guide RNA	No. of hairy roots examined	No. of hairy roots with mutations	Mutation rates (%)	Indel frequencies (%)
*bar*	*bar*-SP1	30	11	36.7	1.3~21.0
	*GmFEI2*-SP1	30	18	60.0	0.6~18.8
*GmFEI2*	*GmFEI2*-SP2	30	28	93.3	1.0~95.0
	*GmFEI2*-SP3	30	3	10.0	5.4~27.0
*GmFEI1*	*GmFEI2*-SP3	30	3	10.0	20.3~32.7
	*GmSHR*-SP1	30	15	50.0	2.3~21.3
*GmSHR*	*GmSHR*-SP2	22	10	45.4	8.7~30.0
	*GmSHR*-SP3	28	10	35.7	2.8~28.7

Mutation rates (%) = (No. of hairy roots with mutations / No. of hairy roots examined) x 100%. Indel frequencies were calculated by measuring the band intensities using the ImageJ gel quantification software.

### CRISPR/Cas9-induced Mutations in Soybean Endogenous Genes

To determine the capacity and efficiency of the CRISPR/Cas9 system for inducing mutations in endogenous soybean genes, six sgRNAs were designed to edit different regions of two endogenous genes (*GmFEI2* and *GmSHR*) in soybean hairy roots. PCR/RE assays were conducted to detect mutations in *GmFEI2*-SP1, *GmFEI2*-SP2, *GmFEI2*-SP3 and *GmSHR*-SP3 target regions. Each of these sgRNAs had a restriction enzyme target site at the Cas9 cleavage site (*Alu*I for *GmFEI2*-SP1, *Bsl*I for *GmFEI2*-SP2 and *GmFEI2*-SP3, *Eco*47I for *GmSHR*-SP3), 3 bp before the protospacer adjacent motif (PAM). Mutations in the *GmFEI2*-SP1 site were identified in 18 of the 30 independent transgenic hairy roots, with indel frequencies ranging from 0.6% to 18.8%, as estimated by band intensities (S1B Fig). Mutations in *GmFEI2*-SP2 site were identified in 28 of the 30 independent transgenic hairy roots, with indel frequencies ranging from 1.0% to 95.0% (Fig 2B), and mutations in the *GmSHR*-SP3 site were identified in 10 of the 28 independent transgenic hairy roots with indel frequencies ranging from 2.8% to 28.7% (S4B Fig). Cloning and sequencing of these undigested products revealed various indel types in all targeted sites (S1C and S4C Figs, Fig 2C). There was no appropriate restriction enzyme site in the *GmSHR*-SP1 and *GmSHR*-SP2 target sequences at the Cas9 cleavage site. As an alternative, T7EI assay can be used. These experiments showed that mutations in *GmSHR*-SP1 site were identified in 15 of the 30 independent transgenic hairy roots, with indel frequencies ranging from 2.3% to 21.3%, as estimated by band intensities (S2B Fig), and mutations in *GmSHR*-SP2 site were identified in 10 of the 22 independent transgenic hairy roots, with indel frequencies ranging from 8.7% to 30.0% (S3B Fig). Cloning and sequencing of these PCR products confirmed the presence of indel mutations at the two target sites (S2C and S3C Figs).

### Multiple Endogenous Gene Targeting with CRISPR/Cas9

To detect the editing potential of the CRISPR/Cas9 system for more than one gene with only one customized sgRNA at one time, we designed a sgRNA, namely *GmFEI2-SP3*. Both *GmFEI1* (*Glyma01g35390*) and *GmFEI2* (*Glyma09g34940*) have this target sequence at the third exon (Fig 3A). PCR/RE assay showed that expression of this sgRNA: Cas9 in soybean hairy roots caused indels at the target sites of both genes at one time, although they had different efficiencies (Fig 3B). Undigested bands were subsequently cloned and sequenced to detect the type of indels in the two genes. Some small insertions and deletions were detected in the target sites (Fig 3C).

## Discussion

The CRISPR/Cas9 system has been successfully used for genome engineering in many important crops. However, this method has not been widely used in soybean. In the present study, we described a rapid and highly specific method for generating and detecting CRISPR/Cas9-mediated genome editing in desired target genes in soybean. To improve transformation efficiency, the sgRNA and Cas9 cassettes were assembled on one vector. The Cas9 was codon-optimized for dicotyledons. Once a desired target sequence was selected, only the DNA sequence encoding sgRNA needs to replace. This vector contained a GFP fluorescent label and greatly improved the efficiency of screening for positive hair roots. The results showed that the CRISPR/Cas9 system could edit both endogenous and exogenous genes in soybean hairy roots. Indeed, we considered that this technology might be useful in soybean whole-plant transformation. Induced hairy root is a rapid model system for studying the CRISPR/Cas9 system in soybean. Testing the effectiveness of target sites in soybean hairy roots before generating transgenic plants can be time saving, less labor intensive and more cost effective.

As previously described, the efficiencies of CRISPR/Cas9-mediated mutations were estimated in crops, such as rice and wheat, and model plants, such as *A*. *thaliana* and *N*. *benthamiana*. The mutagenesis efficiencies of 15~38% and 3~8% were, respectively, detected in rice [7] and wheat [29] using PEG-based protoplast transformation. Rice callus cells were particle bombarded with Cas9 and sgRNA expression plasmids, resulting in mutagenesis efficiencies of 7.1~9.4% [7]. In another study, PEG-based protoplast transformations of *Arabidopsis* and *N*. *benthamiana* targeting various endogenous genes showed mutation efficiencies between 1 and 7% (*Arabidopsis*) or approximately 38% (*N*. *benthamiana*) [30]. In contrast, mutagenesis efficiencies of 2.7 ~ 4.8% were detected in *N*. *benthamiana* leaves after using agroinfiltration [30]. In the present study, the efficiency of each target site was estimated. The CRISPR/Cas9 system presents relatively high mutation rates in soybean hairy roots. Targeted DNA mutations were detected in approximately 54% of the 170 transgenic hairy roots, with indel frequencies ranging from 0.6 to 95.0%. However, we observed that the efficiencies were different between various designed sgRNAs, even in the same gene. As for the target gene *GmFEI2*, the *GmFEI2*-SP2 target site was significantly more efficient for inducing mutations compared with the other two sgRNAs *GmFEI2*-SP1 and *GmFEI2*-SP3. The reasons for this observation remain unknown. In the present study, the sgRNA cassette was driven by the *Arabidopsis* U6 promoter because soybean is a dicotyledonous plant; thus, whether the soybean endogenous U6 promoter could improve the capacity of CRISPR/Cas9 for genome editing needs further research.

In addition to single site or gene targets, the CRISPR/Cas9 system can be used for multiplex gene editing in plant tissues. One sgRNA with two identical target sites in two loci has been shown to generate mutations at two target loci in *Arabidopsis* protoplasts [30]. In the present study, we demonstrated that double gene targeting can be achieved in soybean via simultaneous cleavage at two targeted sites using only one customized sgRNA. Thus, multiple genes could also be targeted with only one customized sgRNA according to the same principle. This method might be useful for the application of CRISPR/Cas9 to soybean and other plant species with duplicated genes. The CRISPR/Cas9 system can successfully be applied in a variety of plant species to simultaneously target more than two genes by increasing the number of sgRNA cassettes in one vector [11, 31]. Large chromosomal segment excision between two distant nuclease-targeted sites using the CRISPR/Cas9 system has previously been reported in rice [15]. These methods can be used in soybean to accelerate the application of the CRISPR/Cas9 system.

The CRISPR/Cas9 system could facilitate the development of additional applications in soybean through homology-directed repair (HDR) using double-stranded DNA (dsDNA) donor templates, for example, to create targeted gene insertions or allelic replacements, both of which have been accomplished in other species [7, 19]. CRISPR/Cas9 has also been used to mediate efficient transcriptional activation in human cells [32]. Hairy roots with characteristics of rapid growth, polytomy and apogeotropism confirm the morphological characteristics of root systems and maintain complete metabolic pathways in physiology. We consider that the multiplex activation of genes that regulate the production of secondary metabolism in soybean hairy roots could provide a method to rapidly produce secondary metabolites.

## Supporting Information

S1 Fig CRISPR/Cas9-induced mutations in the *GmFEI2*-SP1 target site. (A) Schematic illustrating the *GmFEI2*-SP1 target sequence (blue) and corresponding PAM (red). PCR amplification spanning the target loci was conducted using the primers *GmFEI2*-SP1-F and *GmFEI2*-SP1-R. The restriction enzyme *Alu*I at the Cas9 cleave site is underlined. (B) PCR/RE assay to detect CRISPR/Cas9-induced mutations in target loci. Lanes 1–30, PCR products of samples treated with sgRNA: Cas9 were digested with *Alu*I. Lanes WT and WT*, undigested and digested wild-type controls, respectively. The red arrowhead indicates the undigested bands. The numbers at the bottom of the gels indicate mutation frequencies measured according to band intensities. M, DL2000 ladder DNA marker. (C) Cloning and sequencing of the undigested bands. Deletions and insertions are indicated as dashes and red lowercase letters, respectively. The types of mutations are indicated in the right column.
(TIF)

S2 Fig CRISPR/Cas9-induced mutations in the *GmSHR*-SP1 target site. (A) Schematic illustrating the *GmSHR*-SP1 target sequence (blue) and corresponding PAM (red). PCR amplification spanning the target loci was conducted using the primers *GmSHR*-SP1-F and *GmSHR*-SP1-R. (B) T7EI assay to detect CRISPR/Cas9-induced mutations in target loci. Lanes 1–30, PCR products of samples treated with sgRNA: Cas9 were annealed and digested with T7EI. Lanes WT and WT*, undigested and digested wild-type controls, respectively. The red arrowhead indicates the digested bands. The numbers at the bottom of the gels indicate mutation frequencies measured according to band intensities. M, DL2000 ladder DNA marker. (C) Cloning and sequencing of PCR products which have the digested bands. Deletions and insertions are indicated as dashes and red lowercase letters, respectively. The types of mutations are indicated in the right column.
(TIF)

S3 Fig CRISPR/Cas9-induced mutations in the *GmSHR*-SP2 target site. (A) Schematic illustrating the *GmSHR*-SP2 target sequence (blue) and corresponding PAM (red). PCR amplification spanning the target loci was conducted using the primers *GmSHR*-SP2-F and *GmSHR*-SP2-R. (B) T7EI assay to detect CRISPR/Cas9-induced mutations in target loci. Lanes 1–22, PCR products of samples treated with sgRNA: Cas9 were annealed and digested with T7EI. Lanes WT and WT*, undigested and digested wild-type controls, respectively. The red arrowhead indicates the digested bands. The numbers at the bottom of the gels indicate mutation frequencies measured according to band intensities. M, DL2000 ladder DNA marker. (C) Cloning and sequencing of PCR products which have the digested bands. Deletions and insertions are indicated as dashes and red lowercase letters, respectively. Base substitutions are indicated with green capital letters. The types of mutations are indicated in the right column.
(TIF)

S4 Fig CRISPR/Cas9-induced mutations in the *GmSHR*-SP3 target site. (A) Schematic illustrating the *GmSHR*-SP3 target sequence (blue) and corresponding PAM (red). PCR amplification spanning the target loci was conducted using the primers *GmSHR*-SP3-F and *GmSHR*-SP3-R. The restriction enzyme *Eco*47I at the Cas9 cleave site is underlined. (B) PCR/RE assay to detect CRISPR/Cas9-induced mutations in target loci. Lanes 1–28, PCR products of samples treated with sgRNA: Cas9 were digested with *Eco*47I. Lanes WT and WT*, undigested and digested wild-type controls, respectively. The red arrowhead indicates the undigested bands. The numbers at the bottom of the gels indicate mutation frequencies measured according to band intensities. M, DL2000 ladder DNA marker. (C) Cloning and sequencing of the undigested bands. Deletions and insertions are indicated as dashes and red lowercase letters, respectively. Base substitutions are indicated with green capital letters. The types of mutations are indicated in the right column.
(TIF)

S5 Fig Schematic illustrating the basic architecture of the constructs used for CRISPR/Cas9-mediated genome editing. GFP, green fluorescent protein. atU6, *Arabidopsis* U6 promoter. gRNA, guide-RNA. dpCas9, Cas9 codon-optimized for dicotyledons. Ubi promoter, maize ubiquitin promoter.
(TIF)

S6 Fig Characterization of transgenic hairy roots with a GFP fluorescence labeling. (TIF)

S1 File The full sequence of the Cas9 used in the present study. (DOC)

S1 Table Primer sequences used in the present study. (DOC)
