# Supplementary figures and images for: CRISPR/Cas9-Mediated Genome Editing in Soybean Hairy Roots
Source: PLoS One. 2015 Aug 18;10(8):e0136064. doi: 10.1371/journal.pone.0136064 (PMC4540462; doi:10.1371/journal.pone.0136064)

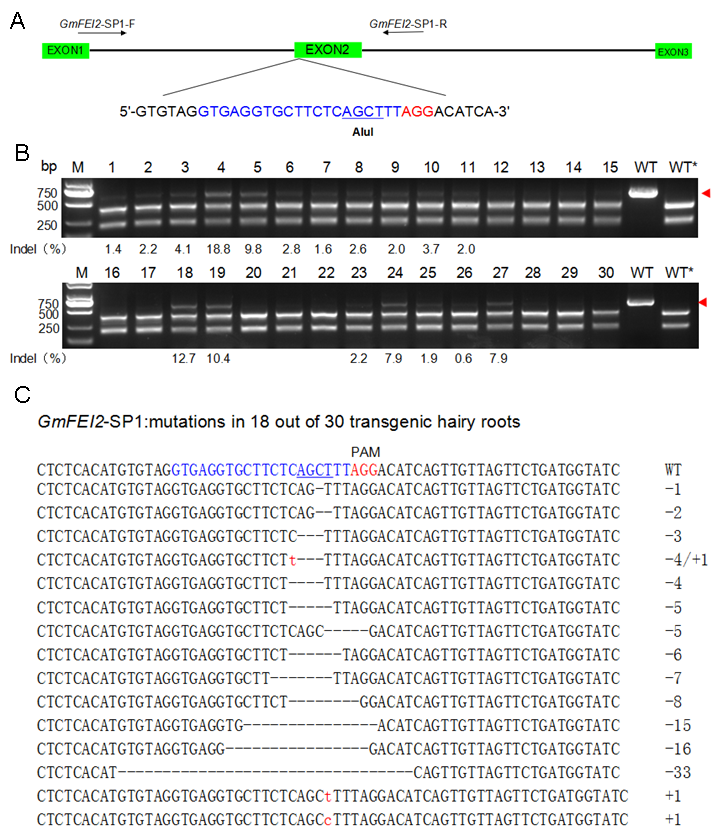

Supplement: S1 Fig — (A) Schematic illustrating the GmFEI2-SP1 target sequence (blue) and corresponding PAM (red). PCR amplification spanning the target loci was conducted using the primers GmFEI2-SP1-F and GmFEI2-SP1-R. The restriction enzyme AluI at the Cas9 cleave site is underlined. (B) PCR/RE assay to detect CRISPR/Cas9-induced mutations in target loci. Lanes 1–30, PCR products of samples treated with sgRNA: Cas9 were digested with AluI. Lanes WT and WT*, undigested and digested wild-type controls, respectively. The red arrowhead indicates the undigested bands. The numbers at the bottom of the gels indicate mutation frequencies measured according to band intensities. M, DL2000 ladder DNA marker. (C) Cloning and sequencing of the undigested bands. Deletions and insertions are indicated as dashes and red lowercase letters, respectively. The types of mutations are indicated in the right column. (TIF) [file pone.0136064.s001.tif]

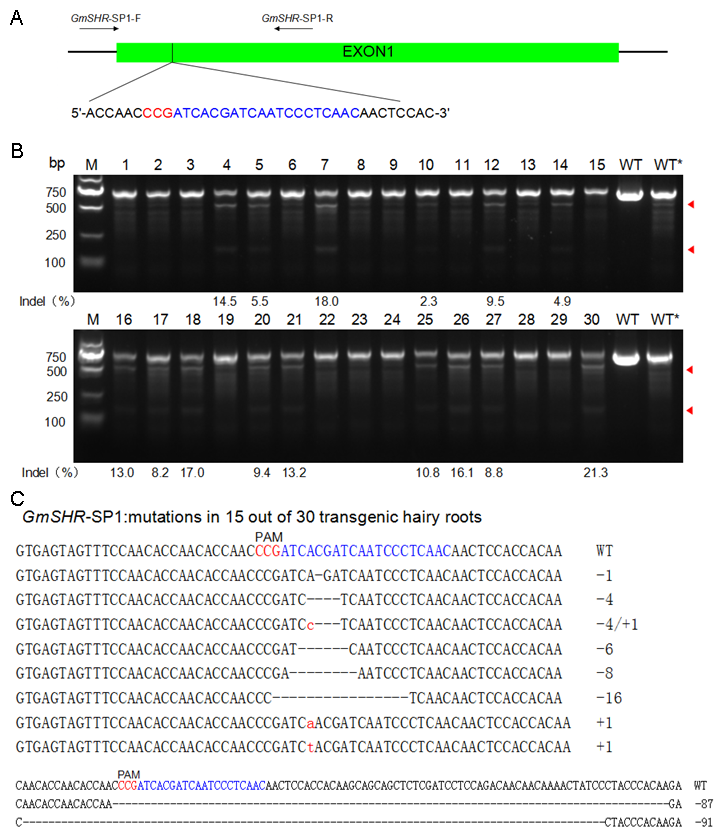

Supplement: S2 Fig — (A) Schematic illustrating the GmSHR-SP1 target sequence (blue) and corresponding PAM (red). PCR amplification spanning the target loci was conducted using the primers GmSHR-SP1-F and GmSHR-SP1-R. (B) T7EI assay to detect CRISPR/Cas9-induced mutations in target loci. Lanes 1–30, PCR products of samples treated with sgRNA: Cas9 were annealed and digested with T7EI. Lanes WT and WT*, undigested and digested wild-type controls, respectively. The red arrowhead indicates the digested bands. The numbers at the bottom of the gels indicate mutation frequencies measured according to band intensities. M, DL2000 ladder DNA marker. (C) Cloning and sequencing of PCR products which have the digested bands. Deletions and insertions are indicated as dashes and red lowercase letters, respectively. The types of mutations are indicated in the right column. (TIF) [file pone.0136064.s002.tif]

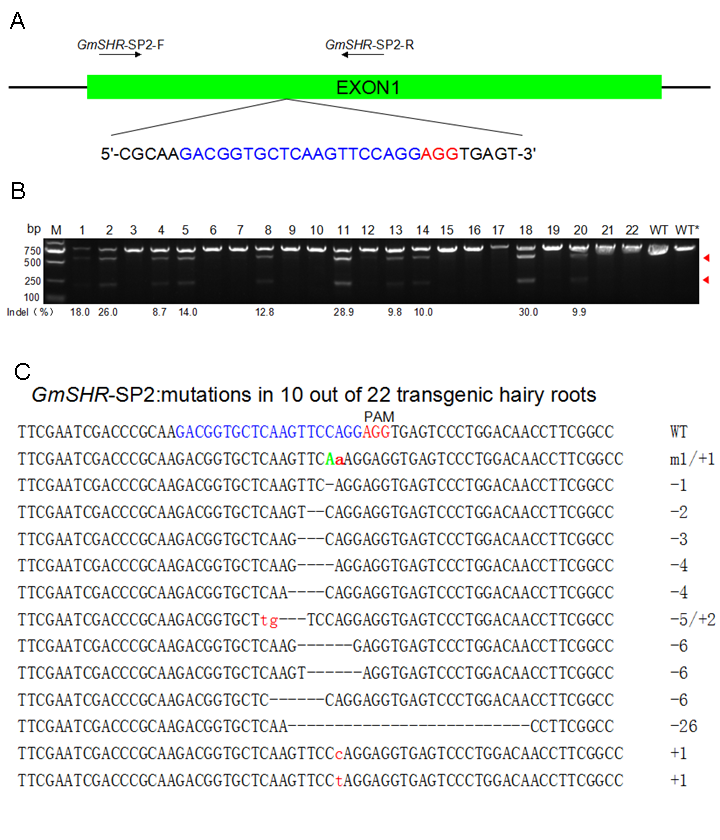

Supplement: S3 Fig — (A) Schematic illustrating the GmSHR-SP2 target sequence (blue) and corresponding PAM (red). PCR amplification spanning the target loci was conducted using the primers GmSHR-SP2-F and GmSHR-SP2-R. (B) T7EI assay to detect CRISPR/Cas9-induced mutations in target loci. Lanes 1–22, PCR products of samples treated with sgRNA: Cas9 were annealed and digested with T7EI. Lanes WT and WT*, undigested and digested wild-type controls, respectively. The red arrowhead indicates the digested bands. The numbers at the bottom of the gels indicate mutation frequencies measured according to band intensities. M, DL2000 ladder DNA marker. (C) Cloning and sequencing of PCR products which have the digested bands. Deletions and insertions are indicated as dashes and red lowercase letters, respectively. Base substitutions are indicated with green capital letters. The types of mutations are indicated in the right column. (TIF) [file pone.0136064.s003.tif]

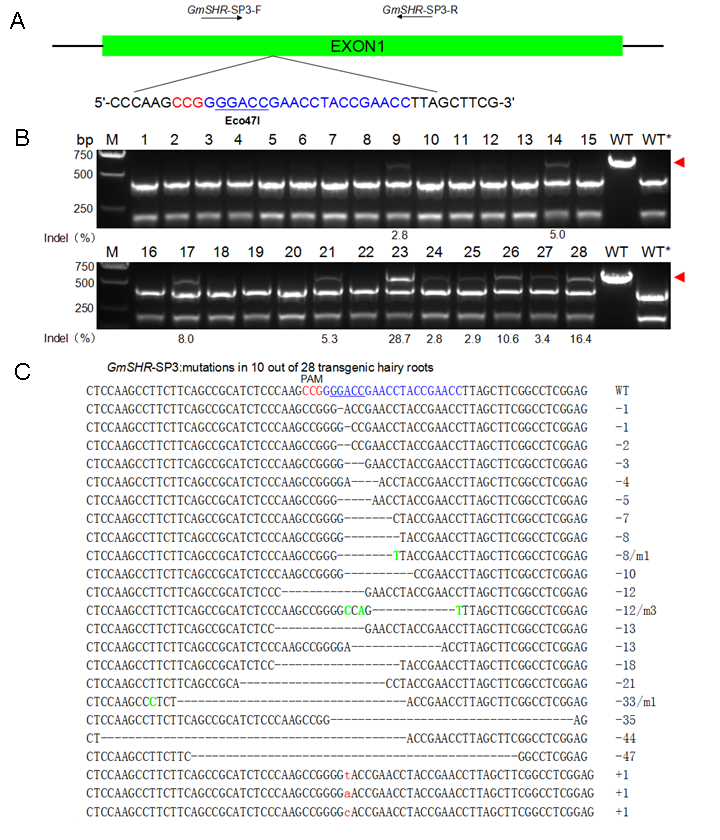

Supplement: S4 Fig — (A) Schematic illustrating the GmSHR-SP3 target sequence (blue) and corresponding PAM (red). PCR amplification spanning the target loci was conducted using the primers GmSHR-SP3-F and GmSHR-SP3-R. The restriction enzyme Eco47I at the Cas9 cleave site is underlined. (B) PCR/RE assay to detect CRISPR/Cas9-induced mutations in target loci. Lanes 1–28, PCR products of samples treated with sgRNA: Cas9 were digested with Eco47I. Lanes WT and WT*, undigested and digested wild-type controls, respectively. The red arrowhead indicates the undigested bands. The numbers at the bottom of the gels indicate mutation frequencies measured according to band intensities. M, DL2000 ladder DNA marker. (C) Cloning and sequencing of the undigested bands. Deletions and insertions are indicated as dashes and red lowercase letters, respectively. Base substitutions are indicated with green capital letters. The types of mutations are indicated in the right column. (TIF) [file pone.0136064.s004.tif]

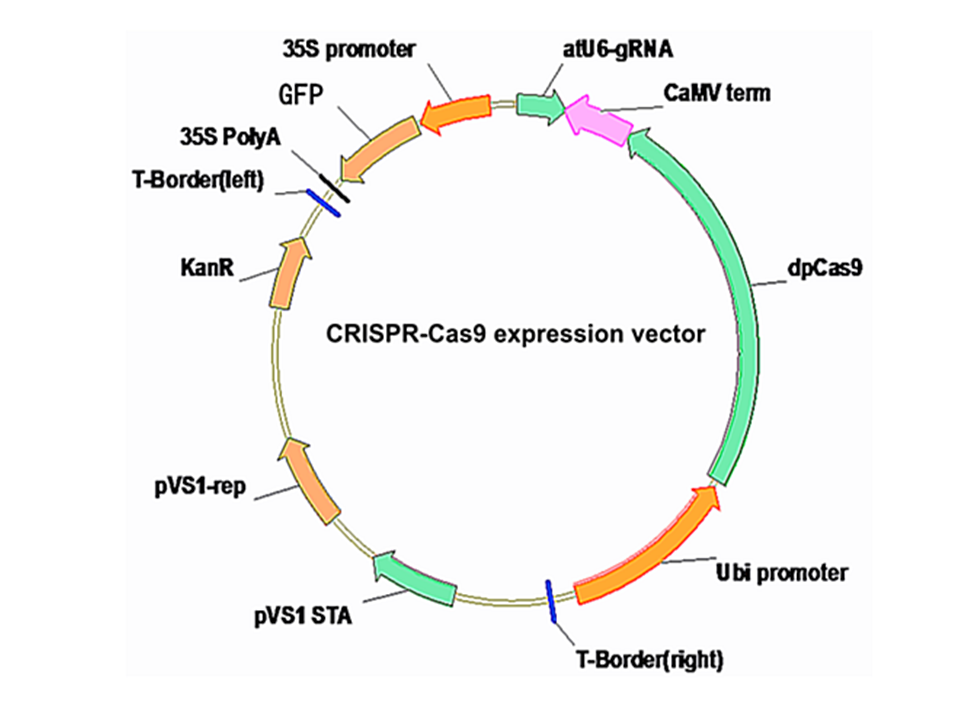

Supplement: S5 Fig — GFP, green fluorescent protein. atU6, Arabidopsis U6 promoter. gRNA, guide-RNA. dpCas9, Cas9 codon-optimized for dicotyledons. Ubi promoter, maize ubiquitin promoter. (TIF) [file pone.0136064.s005.tif]

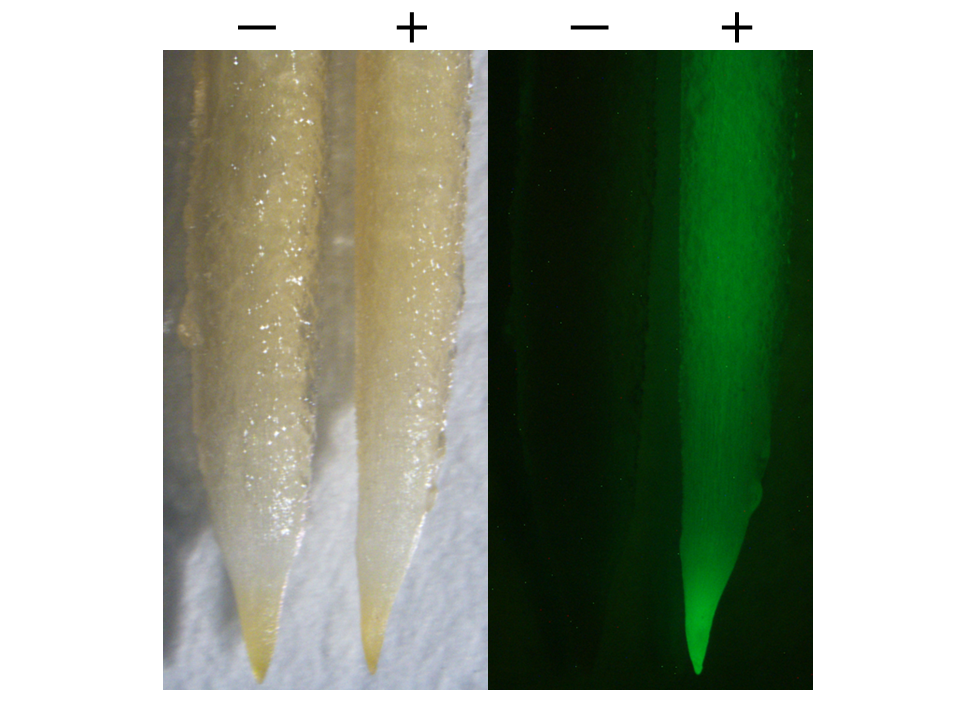

Supplement: S6 Fig — (TIF) [file pone.0136064.s006.tif]
