# Supplementary material for: CRISPR/Cas9-Mediated Genome Editing in Soybean Hairy Roots
Source: PLoS One. 2015 Aug 18;10(8):e0136064. doi: 10.1371/journal.pone.0136064 (PMC4540462; doi:10.1371/journal.pone.0136064)
Supplement: S1 File — (DOC) [file pone.0136064.s007.doc]

# The full DNA sequence of the Cas9

atggcccctaagaagaagagaaaggtcggtattcacggcgttcctgcggcgatggacaagaagtatagtattggtctggacattgggacgaattccgttggctgggccgtgatcaccgatgagtacaaggtcccttccaagaagtttaaggttctggggaacaccgatcggcacagcatcaagaagaatctcattggagccctcctgttcgactcaggcgagaccgccgaagcaacaaggctcaagagaaccgcaaggagacggtatacaagaaggaagaataggatctgctacctgcaggagattttcagcaacgaaatggcgaaggtggacgattcgttctttcatagattggaggagagtttcctcgtcgaggaagataagaagcacgagaggcatcctatctttggcaacattgtcgacgaggttgcctatcacgaaaagtaccccacaatctatcatctgcggaagaagcttgtggactcgactgataaggcggaccttagattgatctacctcgctctggcacacatgattaagttcaggggccattttctgatcgagggggatcttaacccggacaatagcgatgtggacaagttgttcatccagctcgtccaaacctacaatcagctctttgaggaaaacccaattaatgcttcaggcgtcgacgccaaggcgatcctgtctgcacgcctttcaaagtctcgccggcttgagaacttgatcgctcaactcccgggcgaaaagaagaacggcttgttcgggaatctcattgcactttcgttggggctcacaccaaacttcaagagtaattttgatctcgctgaggacgcaaagctgcagctttccaaggacacttatgacgatgacctggataaccttttggcccaaatcggcgatcagtacgcggacttgttcctcgccgcgaagaatttgtcggacgcgatcctcctgagtgatattctccgcgtgaacaccgagattacaaaggccccgctctcggcgagtatgatcaagcgctatgacgagcaccatcaggatctgacccttttgaaggctttggtccggcagcaactcccagagaagtacaaggaaatcttctttgatcaatccaagaacggctacgctggttatattgacggcggggcatcgcaggaggaattctacaagtttatcaagccaattctggagaagatggatggcacagaggaactcctggtgaagctcaatagggaggaccttttgcggaagcaaagaactttcgataacggcagcatccctcaccagattcatctcggggagctgcacgccatcctgagaaggcaggaagacttctacccctttcttaaggataaccgggagaagatcgaaaagattctgacgttcagaattccgtactatgtcggaccactcgcccggggtaattccagatttgcgtggatgaccagaaagagcgaggaaaccatcacaccttggaacttcgaggaagtggtcgataagggcgcttccgcacagagcttcattgagcgcatgacaaattttgacaagaacctgcctaatgagaaggtccttcccaagcattccctcctgtacgagtatttcactgtttataacgaactcacgaaggtgaagtatgtgaccgagggaatgcgcaagcccgccttcctgagcggcgagcaaaagaaggcgatcgtggaccttttgtttaagaccaatcggaaggtcacagttaagcagctcaaggaggactacttcaagaagattgaatgcttcgattccgttgagatcagcggcgtggaagacaggtttaacgcgtcactggggacttaccacgatctcctgaagatcattaaggataaggacttcttggacaacgaggaaaatgaggatatcctcgaagacattgtcctgactcttacgttgtttgaggatagggaaatgatcgaggaacgcttgaagacgtatgcccatctcttcgatgacaaggttatgaagcagctcaagagaagaagatacaccggatggggaaggctgtcccgcaagcttatcaatggcattagagacaagcaatcagggaagacaatccttgactttttgaagtctgatggcttcgcgaacaggaattttatgcagctgattcacgatgactcacttactttcaaggaggatatccagaaggctcaagtgtcgggacaaggtgacagtctgcacgagcatatcgccaaccttgcgggatctcctgcaatcaagaagggtattctgcagacagtcaaggttgtggatgagcttgtgaaggtcatgggacggcataagcccgagaacatcgttattgagatggccagagaaaatcagaccacacaaaagggtcagaagaactcgagggagcgcatgaagcgcatcgaggaaggcattaaggagctggggagtcagatccttaaggagcacccggtggaaaacacgcagttgcaaaatgagaagctctatctgtactatctgcaaaatggcagggatatgtatgtggaccaggagttggatattaaccgcctctcggattacgacgtcgatcatatcgttcctcagtccttccttaaggatgacagcattgacaataaggttctcaccaggtccgacaagaaccgcgggaagtccgataatgtgcccagcgaggaagtcgttaagaagatgaagaactactggaggcaacttttgaatgccaagttgatcacacagaggaagtttgataacctcactaaggccgagcgcggaggtctcagcgaactggacaaggcgggcttcattaagcggcaactggttgagactagacagatcacgaagcacgtggcgcagattctcgattcacgcatgaacacgaagtacgatgagaatgacaagctgatccgggaagtgaaggtcatcaccttgaagtcaaagctcgtttctgacttcaggaaggatttccaattttataaggtgcgcgagatcaacaattatcaccatgctcatgacgcatacctcaacgctgtggtcggaacagcattgattaagaagtacccgaagctcgagtccgaattcgtgtacggtgactataaggtttacgatgtgcgcaagatgatcgccaagtcagagcaggaaattggcaaggccactgcgaagtatttcttttactctaacattatgaatttctttaagactgagatcacgctggctaatggcgaaatccggaagagaccacttattgagaccaacggcgagacaggggaaatcgtgtgggacaaggggagggatttcgccacagtccgcaaggttctctctatgcctcaagtgaatattgtcaagaagactgaagtccagacgggcgggttctcaaaggaatctattctgcccaagcggaactcggataagcttatcgccagaaagaaggactgggacccgaagaagtatggaggtttcgactcaccaacggtggcttactctgtcctggttgtggcaaaggtggagaagggaaagtcaaagaagctcaagtctgtcaaggagctcctgggtatcaccattatggagaggtccagcttcgaaaagaatccgatcgattttctcgaggcgaagggatataaggaagtgaagaaggacctgatcattaagcttccaaagtacagtcttttcgagttggaaaacggcaggaagcgcatgttggcttccgcaggagagctccagaagggtaacgagcttgctttgccgtccaagtatgtgaacttcctctatctggcatcccactacgagaagctcaagggcagcccagaggataacgaacagaagcaactgtttgtggagcaacacaagcattatcttgacgagatcattgaacagatttcggagttcagtaagcgcgtcatcctcgccgacgcgaatttggataaggttctctcagcctacaacaagcaccgggacaagcctatcagagagcaggcggaaaatatcattcatctcttcaccctgacaaaccttggggctcccgctgcattcaagtattttgacactacgattgatcggaagagatacacttctacgaaggaggtgctggatgcaacccttatccaccaatcgattactggcctctacgagacgcggatcgacttgagtcagctcgggggggataagagaccagcggcaaccaagaaggcaggacaagcgaagaagaagaagtag

# The full protein sequence of the Cas9

MAPKKKRKVGIHGVPAAMDKKYSIGLDIGTNSVGWAVITDEYKVPSKKFKVLGNTDRHSIKKNLIGALLFDSGETAEATRLKRTARRRYTRRKNRICYLQEIFSNEMAKVDDSFFHRLEESFLVEEDKKHERHPIFGNIVDEVAYHEKYPTIYHLRKKLVDSTDKADLRLIYLALAHMIKFRGHFLIEGDLNPDNSDVDKLFIQLVQTYNQLFEENPINASGVDAKAILSARLSKSRRLENLIAQLPGEKKNGLFGNLIALSLGLTPNFKSNFDLAEDAKLQLSKDTYDDDLDNLLAQIGDQYADLFLAAKNLSDAILLSDILRVNTEITKAPLSASMIKRYDEHHQDLTLLKALVRQQLPEKYKEIFFDQSKNGYAGYIDGGASQEEFYKFIKPILEKMDGTEELLVKLNREDLLRKQRTFDNGSIPHQIHLGELHAILRRQEDFYPFLKDNREKIEKILTFRIPYYVGPLARGNSRFAWMTRKSEETITPWNFEEVVDKGASAQSFIERMTNFDKNLPNEKVLPKHSLLYEYFTVYNELTKVKYVTEGMRKPAFLSGEQKKAIVDLLFKTNRKVTVKQLKEDYFKKIECFDSVEISGVEDRFNASLGTYHDLLKIIKDKDFLDNEENEDILEDIVLTLTLFEDREMIEERLKTYAHLFDDKVMKQLKRRRYTGWGRLSRKLINGIRDKQSGKTILDFLKSDGFANRNFMQLIHDDSLTFKEDIQKAQVSGQGDSLHEHIANLAGSPAIKKGILQTVKVVDELVKVMGRHKPENIVIEMARENQTTQKGQKNSRERMKRIEEGIKELGSQILKEHPVENTQLQNEKLYLYYLQNGRDMYVDQELDINRLSDYDVDHIVPQSFLKDDSIDNKVLTRSDKNRGKSDNVPSEEVVKKMKNYWRQLLNAKLITQRKFDNLTKAERGGLSELDKAGFIKRQLVETRQITKHVAQILDSRMNTKYDENDKLIREVKVITLKSKLVSDFRKDFQFYKVREINNYHHAHDAYLNAVVGTALIKKYPKLESEFVYGDYKVYDVRKMIAKSEQEIGKATAKYFFYSNIMNFFKTEITLANGEIRKRPLIETNGETGEIVWDKGRDFATVRKVLSMPQVNIVKKTEVQTGGFSKESILPKRNSDKLIARKKDWDPKKYGGFDSPTVAYSVLVVAKVEKGKSKKLKSVKELLGITIMERSSFEKNPIDFLEAKGYKEVKKDLIIKLPKYSLFELENGRKRMLASAGELQKGNELALPSKYVNFLYLASHYEKLKGSPEDNEQKQLFVEQHKHYLDEIIEQISEFSKRVILADANLDKVLSAYNKHRDKPIREQAENIIHLFTLTNLGAPAAFKYFDTTIDRKRYTSTKEVLDATLIHQSITGLYETRIDLSQLGGDKRPAATKKAGQAKKKK
