# Supplementary material for: CRISPR/Cas9-Mediated Genome Editing in Soybean Hairy Roots
Source: PLoS One. 2015 Aug 18;10(8):e0136064. doi: 10.1371/journal.pone.0136064 (PMC4540462; doi:10.1371/journal.pone.0136064)
Supplement: S1 Table — (DOC) [file pone.0136064.s008.doc]

**S1 Table. Primer sequences used in the present study.**

| Primer | Sequence |
| --- | --- |
| *bar*-F | GCACCATCGTCAACCACTACATC |
| *bar*-R | CAGAAACCCACGTCATGCCAGTT |
| *GmFEI2*-SP1-F | AGCAATCACCCCCGATGGTA |
| *GmFEI2*-SP1-R | AGATGTGACAAACTACATCAGTCAA |
| *GmFEI2*-SP2-F | AGGCTTTCTTTTCTGCCACAATACT |
| *GmFEI2*-SP2-R | TATAAAAATACCAAACGGCATAACG |
| *GmSHR*-SP1-F | CGATTCCCACCGTTAAAAACACG |
| *GmSHR*-SP1-R | CTCCTGGAACTTGAGCACCGTCT |
| *GmSHR*-SP2-F | AGTAGTTTCCAACACCAACACCA |
| *GmSHR*-SP2-R | CGACGGAGGTTAAACGGAGG |
| *GmSHR*-SP3-F | GCGACTTTGAATTCTCCGGC |
| *GmSHR*-SP3-R | GCCAAGGCCTCGTCTTCTTT |
| *GmFEI1*-F | GATTTTGTGAATAAATTTATTGTCT |
| *GmFEI1*-R | ACTCTGGTGGAATTGACCCATAGAA |
| *GmFEI2*-F | TGCTTAATTCGATTTTTTTTACC |
| *GmFEI2*-R | ATTGTCCCATAGAAGTTGTTGTT |
